# Methods of Meat-inspection1Read before the Keystone Veterinary Medical Association, November, 1897.

**Published:** 1898-01

**Authors:** Leonard Pearson

**Affiliations:** Veterinary Department, University of Pennsylvania


THE JOURNAL

OF

COMPARATIVE MEDICINE AND

VETERINARY ARCHIVES.

Vol. XIX. *JANUARY, 1898.* No. 1.

### METHODS OF MEAT-INSPECTION.[1]

By Leonard Pearson, B.S., V.M.D.,

VETERINARY DEPARTMENT, UNIVERSITY OF PENNSYLVANIA.

It is not necessary to submit to this audience any argument to prove the importance of meat as an article of diet. This is a matter that is so thoroughly understood and universally recognized that it may be accepted as axiomatic that meat is not only essential as food, but that the activity of a people is indicated largely by the amount of flesh consumed. In 1890 the British Government published a table showing the amount of meat used in the different civilized lands. This table supports the statement just made. The amounts consumed per capita and per annum are as follows :

| | | | |
|---|---|---|---|
| Australia | 111.6 kg. | Belgium and Holland | 31.3 kg. |
| United States | 54.4 " | Austria and Hungary | 29.0 " |
| Great Britain | 47.6 " | Russia | 21.8 " |
| Sweden and Norway | 39.5 " | Spain | 22.2 " |
| France | 33.6 " | Italy | 10.4 " |
| Germany | 31.6 " | | |

It will be seen that the amount consumed in the United States is greater than in any other part of the world, with the exception of Australia, where meat is so very cheap that only the more desirable portions are used as food, and the actual consumption is less than the figures indicate.

Since flesh enters so largely into our diet, and since it is derived from animals that undergo the same disease-processes that we do, and is composed of such fragile compounds that it takes on irritant and toxic properties very quickly, unless handled with the

[1] Read before the Keystone Veterinary Medical Association, November, 1897.

165752

greatest care, it is not surprising to learn that the control of this food has attracted the attention of sanitarians from the earliest times. The first meat-inspection was under the control of ecclesiastical authorities, and in some sects, as the Mohammedans and Jews, it is carried out under the same direction to this day. Such an inspection, when originated, was as careful and thorough as the knowledge of the times would permit, but the regulations under which it is now enforced do not represent the most useful and efficient methods.

In the last century the general public was aroused, and many of the countries of Continental Europe passed laws providing for the inspection of meat, and some municipalities erected abattoirs, where all of the slaughtering of the city should be carried on under competent supervision. More recently, since the bacterial origin of many diseases has been demonstrated and the close relationship of many of the diseases of man and animals has been established, the importance of rational meat-inspection has been greatly emphasized. At this time all of the countries of Continental Europe and the British Isles have a system of meat-inspection, which, although incomplete in some places and in some details, is in the main sufficient to protect the consumer from the numerous maladies that may be contracted by eating the flesh of diseased animals, or meat that has been improperly cared for or preserved.

The system of meat-inspection that has proven most satisfactory in Europe includes the establishment of a municipal abattoir under the direction of a veterinarian trained in meat-inspection, and it is provided that all animals killed locally shall be slaughtered in this establishment. All meat-producing animals are examined while living and after they are killed. Both inspections are made because there are some conditions that cannot well be detected after the animal is slaughtered, but often have an important effect on the quality of the meat, as fever, fatigue, exhaustion, starvation, and excitement. After an animal is slaughtered its organs and flesh are examined for evidences of diseases that are directly transmissible to the consumer, parasites that may be injurious, and other conditions which, while not directly transmissible in themselves, render the flesh indigestible or toxic, and thus produce digestive disturbances or more serious disease. The carcass is also examined for the purpose of discovering conditions that render the meat innutritious or offensive, such as chronic wasting diseases, emaciation, old age, immaturity, and advanced pregnancy. Moreover, the method of slaughtering and handling the dressed meat is supervised, and it is seen to that all of the steps are carried out in a cleanly manner and that the meat is not unnecessarily contaminated by carelessness and filthy surroundings. The conditions and diseases to be sought for and avoided are so numerous that they cannot be discussed at greater length in this paper.

Germany has more than six hundred slaughter-houses belonging to municipalities ; each of these is under the direction of a veterinarian. In most of them there are separate halls for slaughtering the different kinds of meat-producing animals : one each for cattle, sheep, and swine. The butchers in these cities pay a reasonable rental, and are permitted to use all the facilities provided and to enjoy the advantages of buildings equipped with all possible labor-saving devices and modern conveniences. Each slaughter-house has a large cold-storage chamber, which can be used by the individual butchers upon the payment of a small fee. In this way the butchers who kill but a few animals a week, and cannot afford to equip satisfactory establishments of their own, receive all of the benefits enjoyed by those who conduct large businesses. Moreover, their meat goes on the market with the official stamp of the inspector showing that it is wholesome. The meat that is condemned is made into fertilizer by the establishment on the account of the individual butcher, and this part of the business is conducted so well that it is usually possible to realize from 20 to 25 per cent. of the original cost of the animal. This saving cannot be effected under less favorable conditions.

In this country the existing systems of meat-inspection may be divided into two classes, national and local. For some years the United States Bureau of Animal Industry has conducted a constantly-improving meat-inspection service that now extends to animals killed for export and for interstate trade in the principal meat-packing centres of the country. The work is performed by veterinarians, who examine all carcasses, stamp those that are sound, and condemn those that are unfit for food. There is also a microscopical examination of pork for the detection of trichinæ, but this extends only to the products prepared for export. Some of the cities in the United States have also organized more or less complete meat-inspection systems. The system in New Orleans, originated and developed principally by Dr. A. S. Wheeler, is perhaps as perfect as exists anywhere in the United States. It provides that all animals killed locally for food shall be inspected, and the meat is stamped. Moreover, all dressed meat brought into the city must be stamped in a similar way. And it is unlawful for any butcher to sell meat that does not bear the stamp of the meat-inspector. In Montgomery all meat-producing animals are killed in a central slaughter-house under the supervision of a meat-inspector. These systems, and all that are followed in European countries, place the responsibility of deciding whether a given carcass is suitable for food upon an inspector who is trained in animal pathology.

In some other cities, as Philadelphia, the meat-inspection system is based upon an entirely different principle. There are laws prohibiting the sale of diseased or unwholesome meat, and it is assumed that the butcher is always competent to determine this point. Under this system detectives or police-officers are appointed to visit slaughter-houses, markets, and butcher-shops, hunt for diseased or unwholesome meat, which is condemned by a veterinary adviser called in by them, and the seller is often prosecuted. It is scarcely necessary to say that this system is undesirable: First, because it does not include an inspection of all meat sold, and inevitably permits the consumption of much that is injurious; and, second, because it assumes knowledge on the part of the butcher that he cannot possibly possess, and makes him responsible for conditions that he cannot recognize. The system is therefore incomplete, and as a permanent system it is unjust. Its chief advantage lies in the fact that it tends to make butchers more careful, so that gross pathological conditions do not reach their stalls, and a portion of the diseased meat that would otherwise be placed upon the market is barred. However, such a system constitutes a beginning in the right direction, but no municipality should be satisfied with it if a better can be obtained.

Municipal meat-inspection is of more importance in the East than in the West, because tuberculosis is more prevalent in this region, and a great many worn-out dairy-cows are sent to the shambles. Many of these cattle are afflicted with tuberculosis and other chronic ailments. They are frequently emaciated, and constitute the most dangerous class of beef-animals. Philadelphia is situated in the midst of a bountiful dairy-district, and is a large consumer of these animals. They are not killed in a large central abattoir under constant supervision, but in numerous little slaughter-houses scattered throughout the city and its suburbs. There are about one hundred slaughter-houses in Philadelphia. Many of them are quite small, situated on back streets surrounded by stables and dwelling-houses. In these establishments cattle are frequently killed at night, or very early in the morning, and are not inspected at all. Occasionally, and as often as possible, the inspector drops in while the carcasses are being dressed, and his vigilance is rewarded almost daily by the discovery of a diseased and dangerous animal. The business of these slaughter-houses is conducted so irregularly that it is not possible to properly control them without having almost as many meat-inspectors as slaughter-houses, and if the force were enlarged to these dimensions the sanitary conditions and the surroundings of the slaughter-houses would still be such as to seriously injure the wholesomeness and keeping qualities of much of the meat dressed in them.

A further reason for a better system of meat-inspection here is that there is a constant and growing demand for many parts of carcasses which are more frequently diseased than the flesh, and were formerly thrown away. Our ever-increasing foreign population consumes viscera for which there was no market a few years ago, and meat-inspectors frequently find that such organs are diseased to an extent that renders them unwholesome, while the rest of the carcass can safely be sold. As a result of the fact that inspectors are not constantly present, a great many diseased carcasses are unquestionably sold, and frequently without the knowledge of the butcher who handles them. His training is not sufficient to enable him to detect important symptoms and lesions. In some cases, however, he does detect and remove them so thoroughly that the suspicions of the meat-inspector are not aroused.

The conditions that prevail in Philadelphia are not unique. They exist in almost every city in this country, and it is largely on account of the multiplicity of slaughter-houses that thorough systems of meat-inspections have not been more generally established.. An adequate control of the meat-supply of Philadelphia cannot be enforced without a great extension of the present force or a concentration of the business of slaughtering. The latter plan is supported by the experience of all of the older civilized countries, and is to be recommended not only because it would facilitate the inspection of meat, but for several other reasons as well. It would do away with all of the small, poorly equipped, badly managed slaughter-houses, which are in many cases nuisances in their respective neighborhoods. It would make it unnecessary to drive cattle through the streets, a practice that blocks traffic, frightens people, and at times occasions serious accidents. It would give small butchers the advantages enjoyed by wholesalers ; they could use the facilities of the large slaughter-house, which are immeasurably superior to their individual establishments, and the cold-storage system could be used by all, with economy to the dealer and advantage and increased wholesomeness of the meat to the consumer. The offal and the condemned organs and carcasses could be disposed of to better advantage. Local meat would gain in reputation, if such a system were enforced, and trade could be built up on its merits, and competition with Western beef would be less difficult.

Moreover, it has been shown by repeated trials of this system that instead of increasing the cost of meat it tends to reduce it.

A large establishment can be conducted by coöperation between butchers at less expense than when each has his own establishment. In Europe such union or central abattoirs are owned by municipalities, and undoubtedly this is the most desirable system, because under it all butchers are assured equal rights and privileges. It has been found that the rentals derived from these establishments are sufficient not only to pay the running expenses, but to afford a reasonable return for the investment. The whole system is not only of great advantage to the consumer of meats, but it subjects butchers to no hardship whatever, and makes it more convenient and cheaper for them to conduct their trade.

